# Bioactivity and immunological evaluation of LPS from different serotypes of *Helicobacter pylori*

**Published:** 2013-06

**Authors:** Davoud Esmaeilli, Ashraf Mohabati Mobarez, Ali Hatef Salmanian, Ahmad Zavaran Hosseini

**Affiliations:** 1 Department of Bacteriology, Faculty of Medical Sciences, Tarbiat Modares University, Tehran, Iran; 2 Department of Bacteriology, Faculty of Medical Sciences, Baqiyatallah University of Medical Sciences, Tehran, Iran; 3 National Institutes for Genetic Engineering and Biotechnology, Tehran, Iran; 4 Dept. of Immunology, Faculty of Medical Science, Tarbiat Modares University, Tehran, Iran

**Keywords:** LPS, Bioactivity, *H. pylori*

## Abstract

**Background and Objectives:**

*Helicobacter pylori* is the causative agent of peptic ulcer disease and a co-factor in development of gastric malignancies. LPS are among toxic substances produced by *H. pylori* exhibiting low endotoxic activity compared to typical bacterial LPS. The aim of this study was to investigate bioactivity of LPS produced by different serotypes of *Helicobacter pylori* compared to *Escherichia coli* and *Brucella abortus* LPS.

**Materials and Methods:**

Bacterial LPS was extracted by the hot phenol-water method. Biological activities of LPS were determined via the limulus lysate assay, pyrogenic assay, and blood pressure and PBMC induction test in rabbits.

**Results:**

Biological activity of O_2_ serotype LPS of *H. pylori* was less than the biological activity of other *H. pylori* serotypes.

**Conclusion:**

Our data supported the hypothesis that the unique bacterial LPS of the O_2_ serotype must be included in the formulation of a multivalent *H. pylori* vaccine.

## INTRODUCTION

Lipopolysaccharides (LPS) of Gram negative bacteria are major surface antigens that play important roles in the stability of bacterial outer membrane. LPS of *Helicobacter pylori* are structurally similar to LPS of Gram negative bacteria (1). However, lipid A of *H. pylori* is distinct from enterobacteriaceae LPS (2). LPS of *H. pylori* are characterized by fewer and longer fatty acid residues, absence of 4-phosphate groups and an ethanolamine group linked to the glycosyl phosphate; therefor, *H. pylori* LPS shows low biological activity by induction of cytokines (3–8). The endotoxicity, lethality and pyrogenicity activities of *H. pylori* LPS are weaker than other typical LPS such as those from *E. coli* (2, 9–14). It induces LPS such as apoptosis of epithelial cells and gastritis in mice (15, 16).

In this study, we aimed to study the efficacy of *H. pylori* LPS as a vaccine candidate by studying its biological activity using the limulus lysate assay, pyrogenic assay, blood pressure test and PBMC induction test in rabbits.

## MATERIALS AND METHODS

### Bacterial strains and culture

*Helicobacter pylori* Sydney strain1 (SS1) donated by Dr. Kuster (Utrecht University) and serotype O_2_ (donated by Dr. Kuster and Dr. Graham (Baylor College of Medicine) and clinical isolates from Iran hospital were grown in Brucella agar (Merck, Germany) supplemented with defibrinated sheep blood (5%), vancomycin (10 mg/liter), polymyxin B (0.33 mg/liter), and amphotericin B (5 mg/liter). All *H. pylori* cultures were incubated in a CO_2_ incubator (Memmert, Germany) at 37°C with a gas mixture of 10% CO_2_. *H. pylori* strains were identified based on Gram staining, morphology, urease, catalase and oxidase tests. Escherichi coli strain O55:B5 and *Brucella abortus* S99 were cultured in BHI and brucella agar respectively (17, 18).

### Large-scale growth of *H. pylori* for LPS purification

*H. pylori* strains were grown on Brucella agar for 48-72h and then transferred into Brucella broth to reach their mid- log phase (OD_600_:0.4-0.6). The cells were sedimented by cold centrifugation (4°C, 30 min) at 18000 rpm. Bacterial pellets were resuspended in sterile phosphate buffered saline (PBS, pH = 7.4).

A 150 ml aliquot of collected Brucella broth cultures were inoculated into 1 L of *H. pylori* culture medium, at an optimum optical density of 0.6 and incubated for 48 h in a shaker incubator. Finally, the bacterial contents were killed by the addition of 2% (w/v) phenol (20 minute, 15°C), and the bacterial cell debris were separated by centrifugation at 5000×g (4°C, 30 min), and the bacterial pellets were freeze-dried (19–21).

### LPS extraction and silver staining

LPS *of H. pylori* strain SS1, clinical isolates; *H. pylori* serotype O_2_, *E. coli* O55:B5 and *Brucella abortus* S99 were extracted by hot phenol-water method (21). Bacterial colonies were collected in 10 ml of PBS, sonicated (10 cycle, 40s) and mixed with same volume of hot phenol-water (9:1, v: v). The mixture were shaken for 30 min at 65-70rpm. After centrifugation (3500 rpm, 30 min, 4°C) aqueous phases were collected; this step was repeated 3 times. All collected aqueous phases were dialyzed against distilled water for 48 h for the elimination of phenol; liquid phase was saved for B-LPS extraction. The LPSs extracts were concentrated to 1/5 of the initial volume and then digested with RNase H and DNase I (Sigma) at a final concentration of 50 µg /mL at 37°C for 4 h. The digested extract were washed in boiling-water for 15 min and then incubated at 4°C overnight. The obtained supernatants were centrifuged (3000 rpm for 30 min) and dialyzed against distilled water for 48 -72h. Precipitates were collected by centrifugation (5000 rpm, 30 min), suspended in distilled water to remove residual alcohol and dialyzed against distilled water for 48 h. The LPS extracts were centrifuged (100,000×g for 2 h) (16, 22, 18) and the pellets were dialyzed in distilled water and lyophilized.

H-LPS, E-LPS and B- LPS were dissolved in pyrogen free water. SDS-PAGE and silver staining was applied to investigate the electrophoresis pattern of the LPS (23). This pattern was compared with that of *E. coli* O55:B5 and *Brucella abortus* S99 LPS.

### LAL assay

The E-TOXATE reagent kit (Sigma) was used for the LAL assay according to the manufacturer's instruction (Sensitivity = ± 1 ng/mL *E. coli O55:B5* LPS). Pyrogenic free water and standard LPS of *E. coli* were used as the negative and positive controls, respectively (18).

### LPS pyrogeneicity assay in rabbit

Anal temperatures for New Zealand rabbits (3.0 ± 0.2 kg), was measured at 15 min intervals for 1-3h. Rabbits with a temperature variation range of 0.2 or lower were selected. Three groups each containing 3 rabbits were inoculated as follows: group one were injected through marginal ear vein with 0.5 ml normal saline containing H-LPS, B-LPS or E-LPS at a dosage of 100 µg/kg. Negative control group were injected with an equal volume of pyrogen-free normal saline. Anal temperatures were measured in all tested groups. In a pyrogenic material, the temperature degree was lower than 0.5 in one rabbit and total temperature in three rabbits were lower than 1.2 (18).

### Abnormal toxicity test

Balb/c mice, 6-8 weeks weighting 17± 3g, were randomly divided into 6 groups, each group containing 6 animals. Each mice was intraperitoneally injected with 0.3 ml pyrogen-free normal saline containing H-LPS, E-LPS or B-LPS at dosages of 0.3, 0.6, 0.12 mg, respectively. Each of the control mice were intraperitoneally injected with 0.2 ml pyrogen-free saline and all groups were observed for 6 days (18).

### Immunizations and experimental study

Three groups of mice (N = 5/group) were immunized three times intramuscularly (IM) at 10 day intervals. IM immunizations were performed with 10 µg LPS (Group 1) or 10 µg LPS and 10 µg C_P_G oligonucleotides adjuvant (Group 2). IM immunizations were performed into the right thigh. Control animals (Group 3) received the same volume of PBS through the route and schedule (Table 3). Serums were collected 7 days after IM immunizations. Serum IgG1, and IgG2α antibodies specific to *H. pylori* LPS and IFNγ were measured by ELISA.

## RESULTS

**Fig. 1:**
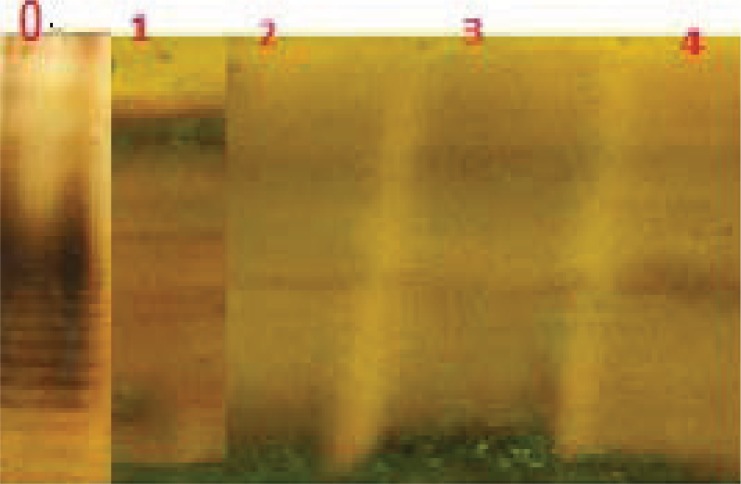
Pattern of LPS in Different serotype of *H. pylori* LPS Lane 1: LPS of *B. abortus* S99, Lane 2 LPS of *H. pylori* ATCC 26695 Lane 3: *H. pylori* serotype O_2_, Lane 4: *H. pylori* SS1, Lane 0 *E. coli* O55:B5.

*H. pylori* LPS were analyzed with 14% SDS-PAGE gels containing 4M urea (Fig. 1) and stained with silver stain, resulting in patterns similar to the observed previously for the LPS of *H. pylori* (24–26).

The extracted LPS had a ladder shaped electrophoretic pattern and the bands were located in three groups: high, medium and low molecular weights.

**Fig. 2:**
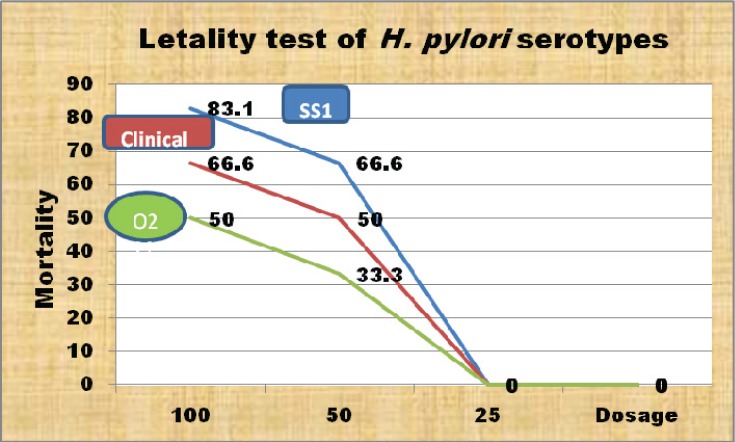
Mouse Lethality test for *H. pylori* serotype.

**Fig. 3:**
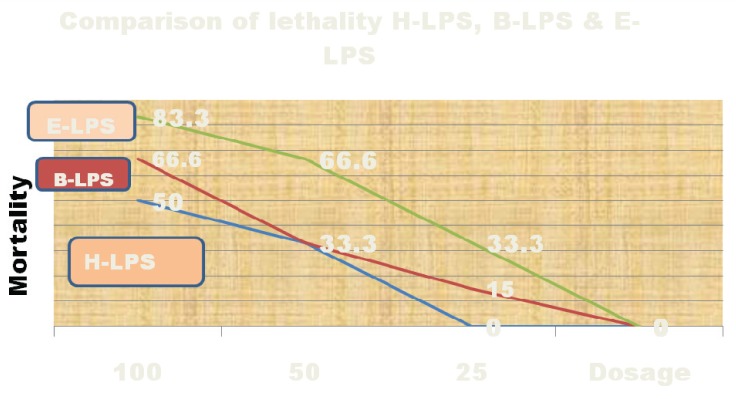
Comparison of lethality for H-LPS,B-LPS & E-LPS.

Pyrogenicity of LPS was tested in rabbits. The temperature for one group rabbits was ≥ 0.5°C and total temperature for rabbits in other group was ≥ 1.2°C. The coagulation ability for *H. pylori* LPS, E-LPS and B-LPS in the LAL assay was as low as 0.75 ng/ml (Table 1). Lethality of H-LPS in mice was significantly lower than E-LPS and B-LPS. B-LPS was weaker than E-LPS (Fig. 2). The lethality of LPS in O_2_ serotype was also weaker than *H. pylori SS1* and the clinical isolates (Fig. 3). The ratio IgG1/IgG2α in the mice immunized with non-detoxified E-LPS and LPS plus CpG was less than 1, while the ratio was ≥1 more than 1 for the control group. LPS of *H. pylori* also induced IFNγ (Table 2).

**Table 1:** Limulus lysate assay of *H. pylori*, *B. abortus* and *E. coli* LPS (ng/ml).

Group	12	6	3	1.5	0.75	0.375	0.1875	0.09745
H-LPS	+	+	+	+	+	_	_	_
E-LPS	+	+	+	+	+	_	_	_
B-LPS	+	+	+	+	+	_	_	_
A pyrogen water	_	_	_	_	_	_	_	_

**Table 2:** Experimental groups of immunized mice and immune responses.

Group	Immunization	IFN-Y^1^	IgG1/IgG2α
1	LPS	0.4±205	0.3±0.63
2	CPG + LPS	0.2±259	0.2±0.57
3	Control	0.4±137	0.4±1.02

1 Mean (±SEM) cytokine and antibody titers assayed by ELISA

## DISCUSSION

Lipopolysaccharides are a family of glycophospholipids that are found in the outer membrane of Gram negative bacteria and are generally toxic with potent immuno-modulating and immune-stimulating properties (27). Clinical isolates of *H. pylori* produce smooth forms of LPS with O-polysaccharide chains of relatively constant chain length compared with enterobacterial LPS (27). LPS is an important pathogenic and virulence factor of *H. pylori*. Previous studies on the bioactivities of *H. pylori* LPS revealed significantly lower endotoxic and immunological activities in compared with enterobacterial LPS. For example, pyrogeneicity and mitogenicity *of H. pylori* LPS was reported to be 1000 fold lower, lethal toxicity in mice was 500 fold lower, and induction of various cytokines was 1000-fold lower than entrobactrial LPS (1, 13).

Bacterial endotoxin possesses broad biological activities and its toxicity is mainly dependent on lipid A (13). Biological activity of LPS such as LAL test, pyrogeneicity and lethality are important factors (13, 14). In this study, we observed that pyrogenicity of *H. pylori* LPS in rabbits and the mortality in mice were less than *E. coli* and *Brucella abortus* LPS's. Also mortality rate in *B-LPS* was less than *E-LPS*. The LAL test results of *H. pylori* LPS were similar to *E. coli* LPS (Table 1). Previous studies showed that *H. pylori* LPS has lower activities as indicated by its lethality and pyrogenicity and the LAL test (3, 13, 18). Different biological activities are conserved in different extraction method for LPS preparation (28, 29). Researchers revealed that *H. pylori* LPS from different strains could be divided into two types: one type with low biological activity and other type with high biological activity (13). These findings revealed the bio-molecular basis for *H. pylori* serotype O_2_ (30). The Lps structure for O_2_ serotype differ from other *H. pylori* LPS in the following aspects: (1) it produces an elongated O-chain polysaccharide and (2) it does not express Lewis blood-group by O chain. These data support the hypothesis that this unique bacterial LPS must be included in the formulation of a multivalent *H. pylori* vaccine.

In this work we aimed at formulating multivalent *H. pylori* LPS-based vaccine by studying LPS's of the different serotypes of *H. pylori*. LPS of the O_2_ serotype was chosen for multivalent vaccine because of less lethality, pyrogenicity in mice and lack of Lewis blood-group expression. The IgG1/IgG2a ratio in the mice immunized with LPS and LPS plus CpG was <1, indicating a Th1 type response, while this ratio was >1 for the control group, indicating a strong Th2 response. These data suggest that immunization with LPS promoted a Th1 immune response and induced IFNγ that is essential for infection clearance (31).
